# Are They Really Lost? “True” Status and Reasons for Treatment Discontinuation among HIV Infected Patients on Antiretroviral Therapy Considered Lost to Follow Up in Urban Malawi

**DOI:** 10.1371/journal.pone.0075761

**Published:** 2013-09-26

**Authors:** Hannock Tweya, Caryl Feldacker, Janne Estill, Andreas Jahn, Wingston Ng’ambi, Anne Ben-Smith, Olivia Keiser, Mphatso Bokosi, Matthias Egger, Colin Speight, Joe Gumulira, Sam Phiri

**Affiliations:** 1 The International Union Against Tuberculosis and Lung Disease, Paris, France; 2 Lighthouse Trust, Lilongwe, Malawi; 3 Institute of Social and Preventive Medicine, University of Bern, Bern, Switzerland; 4 International Training and Education Center for Health, University of Washington, Seattle, Washington, United States of America; 5 Central Monitoring and Evaluation Division / Department for HIV andAIDS, Ministry of Health, Lilongwe, Malawi; 6 Department of Biomedical Informatics, University of Pittsburgh, Pittsburgh, Pennsylvania, United States of America; Infectious Disease Service, United States of America

## Abstract

**Introduction:**

Patients who are lost to follow-up (LTFU) while on antiretroviral therapy (ART) pose challenges to the long-term success of ART programs. We describe the extent to which patients considered LTFU are misclassified as true disengagement from care when they are still alive on ART and explain reasons for ART discontinuation using our active tracing program to further improve ART retention programs and policies.

**Methods:**

We identified adult ART patients who missed clinic appointment by more than 3 weeks between January 2006 and December 2010, assuming that such patients would miss their doses of antiretroviral drugs. Patients considered LTFU who consented during ART registration were traced by phone or home visits; true ART status after tracing was documented. Reasons for ART discontinuation were also recorded for those who stopped ART.

**Results:**

Of the 4,560 suspected LTFU cases, 1,384 (30%) could not be traced. Of the 3,176 successfully traced patients, 952 (30%) were dead and 2,224 (70%) were alive, of which 2,183 (99.5%) started ART according to phone-based self-reports or physical verification during in-person interviews. Of those who started ART, 957 (44%) stopped ART and 1,226 (56%) reported still taking ART at the time of interview by sourcing drugs from another clinic, using alternative ART sources or making brief ART interruptions. Among 940 cases with reasons for ART discontinuations, failure to remember (17%), too weak/sick (12%), travel (46%), and lack of transport to the clinic (16%) were frequently cited; reasons differed by gender.

**Conclusion:**

The LTFU category comprises sizeable proportions of patients still taking ART that may potentially bias retention estimates and misdirect resources at the clinic and national levels if not properly accounted for. Clinics should consider further decentralization efforts, increasing drug allocations for frequent travels, and improving communication on patient transfers between clinics to increase retention and adherence.

## Introduction

As provision of antiretroviral therapy (ART) expands throughout sub-Saharan Africa, the high rate of loss to follow-up (LTFU) among ART patients challenges the long-term success of ART programs. In sub-Saharan Africa, a substantial number of HIV-infected individuals are lost both before [1] and after ART initiation [2,3]. Among those considered LTFU, up to 40% of those traced are reported dead [4]. Adjusting mortality estimates among patients LTFU is recognized as an important correction factor [5] [6]. However, true outcomes of patients considered LTFU do not just include deaths, but may also include those with undocumented transfers, uninterrupted therapy due to antiretroviral drugs (ARVs) received from other sources, and brief treatment discontinuations [7,8]. These additional outcomes are poorly understood.

Although several studies explored reasons for ART discontinuation other than death among LTFU patients in resource-poor countries [9,10], most of these studies were implemented with only small sample sizes and may therefore not be representative [10,11], limiting their generalizability and application to other patient populations or settings. Additional studies using patient data from large, public, urban ART programs may better aid understanding of the reasons for ART discontinuation among ART patients, especially in urban areas. In the current climate of increasing ART program costs and decreasing donor funding, *true* rates of LTFU are even more critical for program evaluation, accurately determining retention in ART care and correctly forecasting usage of HIV-related drugs and commodities.

In 2006, the Lighthouse Trust, the largest ART provider in Malawi’s central region, which has treated more than 38,000 patients between opening in 2001 and present, designed and implemented a Back–to–Care (B2C) program to improve long-term retention on ART at its two urban clinics. The B2C program identifies patients who missed their next scheduled clinic appointment by more than 21 days; these patients are traced and advised to return to the clinic for ART continuation. As a follow-up to our previous papers that determined the proportion of LTFU returned to care through our B2C active tracing [7] and identified the patient characteristics associated with successful tracing [12], we now describe the extent to which patients considered LTFU are misclassified as true disengagement from care when they are still alive on ART and explore the reasons for ART discontinuation among those who actually stop to further improve ART retention programs and policies.

## Methods

### Study design, sites and population

We conducted a retrospective cohort study based on routine data from a patient tracing program at Lighthouse Trust’s two public ART clinics in urban Malawi, Lighthouse and Martin Preuss Center (MPC). All confirmed LTFU patients were traced by either phone or home visit. Lighthouse and MPC ART clinics are located in Lilongwe, Malawi’s capital city. On average, Lighthouse and MPC manage 8,300 and 11,200 patients a month, respectively, using a real-time, point of care electronic data system (EDS) from which the data for this study came [13].

All HIV-infected individuals aged 15 years or older who received ART at Lighthouse and MPC between January 2006 and December 2010 were eligible for inclusion in this study.

### Data collection

As is routine practice at Lighthouse and MPC, during the study period all HIV diagnosed individuals were registered in the EDS and asked for consent at registration to be traced in case of LTFU. HIV-infected adults were started on ART if their CD4 count was equal to or below 250 cells/µl or they were in WHO clinical stage 3 or 4 in accordance with Malawi National ART guidelines during the study period [14]. During the study period, one generic fixed dose combination of treatment (stavudine, lamivudine, nevirapine) was used as first line regimen. Routine return visits were scheduled monthly during the first six months on ART and every two months thereafter if no clinical complications occurred. At each ART visit, adherence was assessed using pill counts and entered in the EDS. The EDS electronically calculated the next scheduled ART visit based on regimen, prescribed schedule, number of tablets newly dispensed, and tablets remaining at present visit. ART program outcomes (alive and on ART, stopped ART, or transferred out) were updated in the EDS at each clinic visit. Deaths were generally updated retrospectively after active tracing.

Data collection for the B2C program was previously described [7]. In brief, patients with any ARV drug dispensing record (including ARV initiation) who missed their next scheduled appointment and, presumably, would have run out of ARVs by three weeks or more were identified using the EDS and considered LTFU. A team of B2C tracers first verified the LTFU list to rule out EDS data errors by cross-checking patient paper files at the clinic. Then, those patients assumed LTFU who consented during ART registration were first traced by phone up to five phone attempts if a working number was provided and/or then up to three home visit attempts if residential information was provided. The reported ART status after either tracing methods was recorded as: 1)dead; 2)transferred to another ART clinic (either ‘official transfer’ if the transfer-out notes were available in the patient’s health passport but not at the ART facility, or ‘self-transfer’ if the patient arranged the transfer independently); 3) alive on ART (with ‘treatment gaps’ if a patient took none or fewer than the prescribed drugs before the interview date (a brief ART interruption), or with ‘uninterrupted therapy’ if the patient was still taking the correct dose of ARVs despite missing appointments); 4) stopped ART (clinician order or self-directed); 5) refused to be interviewed; 6) never started ART although they collected drugs; and 7) LTFU (not traced or not found). We defined a ‘LTFU case’ as a single episode of a missed appointment; patients could appear on the confirmed tracing list more than once during the study period.

For distance to the clinics, we used Euclidian distances in kilometers (Km) between Lighthouse or MPC and patient village or locations of residence determined from Google, Earth.

### Statistical analysis

Statistical analysis was conducted using STATA 12.0. Descriptive statistics was used to explain baseline characteristics of the study population presented. In the analysis of the reasons for collecting ARVs from other sources, we included patients who reported self-transfer to another ART clinic, uninterrupted therapy, treatment gaps and those who stopped taking ARV drugs by themselves. Patients who self-transferred to another ART clinic were included because their experience provided insight into the challenges faced by ART patients, although they successfully remained in care. Similarly, patients who stopped ART provided insight on their experiences before they stopped. Log-binomial regression was used to compare categories of tracing outcomes with baseline characteristics of patients considered LTFU. Tracing outcomes were classified as 1) successfully traced or not traced; 2) Patients who accessed ART from alternative sources or those who did not; 3) Patients with uninterrupted therapy or those who discontinued treatment. Successfully traced, accessing ART from alternative sources and uninterrupted therapy were hazard events. A level of significance of *P* ≤ 0.05 was used.

### Ethical considerations

Consent for tracing was obtained during ART registration according to routine clinic procedures. The study was approved by The Malawi National Health Science Research Committee in Lilongwe and the Ethics Advisory Group of the International Union against Tuberculosis and Lung Disease in Paris, France. The ethics committees waived the need for patient consent to be involved in the study because the study used routine programmatic data and did not include any personal identifiers.

## Results

### Characteristics of the study population

Between January 2006 and December 2010, 21,382 adult HIV-infected individuals accessed ART at Lighthouse and MPC clinics (Table **1**). Of these, 12,384 (58%) were female. The median age at ART start was 34 years (interquartile range (IQR 29-41)). A total of 5,865 patients (27%) started ART in WHO clinical stage 1 or 2 with CD4 count ≤250 cells/µl, 11,677 (55%) in stage 3 and 3,697 (17%) in stage 4. For 145 patients (1%), the reason for starting ART was missing. Maximum and median times of follow-up were 5.0 years and 1.2 years, respectively.

**Table 1:** Baseline characteristics of patients considered lost to follow-up from antiretroviral care at Lighthouse and Martin Preuss Centre Clinics between January 2006 and December 2010.

	**Total (N=4,145)**		**Lighthouse**	**Martin Preuss Centre**
**Characteristics**	**N (%)**		**N (%)**	**N(%)**
Gender				
Male	1,908	(46%)	1,003 (47%)	905 (46%)
Female	2,237	(54%	1,153 (53%)	1,084 (54%)
Age at ART start				
15-24	529	(13%)	234 (11%)	295 (15%)
25-34	1,803	(43%)	865 (40%)	938 (47%)
35-44	1,171	(28%)	692 (32%)	479 (24%)
45+	642	(15%)	365 (17%)	277 (14%)
Reason for starting ART^¥^				
CD4 ≤ 250	881	(22%)	383 (18%)	498 (25%)
WHO Stage 3	2,338	(57%)	1,202 (57%)	1,136 (58%)
WHO Stage 4	878	(21%)	539 (25%)	339 (17%)
Distance to the clinic^£^				
0-5km	1,865	(48%)	835 (41%)	1,030 (54%)
≥5km	2,048	(52%)	1,185 (59%)	863 (46%)
Year of ART start				
<2006	1,121	(27%)	1,054 (49%)	67 (3%)
2007	1,121	(27%)	434 (20%)	687 (35%)
2008	924	(22%)	361 (17%)	563 (28%)
2009-2010	979	(24%)	307 (14%)	672 (34%)

¥ 48 patients did not have information about reason for starting ART; ^£^232 patients did not have information about distance to the clinic

Of all the 21,382 patients, 4,145 (19%) missed their next scheduled clinic appointment by at least 21 days: 3,768 (91%) missed an appointment once, 319 (8%) twice and 58 (1%) at least three times. Median interval between the date the patient missed their appointment and tracing was 1.1 months (IQR 1.0-2.0). Table **1** shows baseline characteristics of patients who missed a scheduled clinic appointment. The majority of these patients were aged between 25 and 34 years (54%), in WHO stage 3 (43%) and lived at least 5km from their clinic (52%). LTFU patients aged 35 years and above or in WHO stage 4 were more likely to be successfully traced (Table **2**). Forty-seven percent of the LTFU patients were successfully traced by phone.

**Table 2:** Comparison of baseline characteristics by tracing outcomes among patients considered lost to follow-up from antiretroviral care at Lighthouse and Martin Preuss Centre Clinics between January 2006 and December 2010.

	**Successfully traced^a^ (n =4,145)**	**Accessed alternative ARV sources^b^ (n= 1,038)**	**Uninterrupted therapy^c^ (n=1,161)**
**Characteristics**	**RR^¥^**	**RR**	**RR**
Gender			
Female	1.00	1.00	1.00
Male	1.01 (0.97-1.05)	1.05 (0.84-1.31)	0.96 (0.77-1.19)
Age at ART start			
15-24	0.89 (0.82-0.96)**	0.64 (0.43-0.96)	0.54 (0.35-0.84)**
25-34	1.00	1.00	1.00
35-44	1.11 (1.06-1.16)***	1.17 (0.91-1.50)	1.16 (0.91-1.47)
45+	1.13 (1.07-1.19)***	0.86 (0.59-1.24)	0.84 (0.58-1.21)
Reason for starting ART			
CD4 ≤ 250	0.96 (0.91-1.01)	0.81 (0.61-1.08)	0.82 (0.62-1.09)
WHO Stage 3	1.00	1.00	1.00
WHO Stage 4	1.06 (1.01-1.11)***	1.08 (0.82-1.43)	1.01 (0.76-1.35)
Distance to the clinic			
< 5km	0.95 (0.91-0.99)	0.99 (0.79-1.24)	1.09 (0.88-1.36)
≥5km	1.00	1.00	1.00
Year of ART start			
<2006	1.00	1.00	1.00
2007	1.04 (0.99-1.10)	0.65 (0.49-0.87)**	0.61 (0.46-0.81)**
2008	0.99 (0.93-1.05)	0.65 (0.48-0.90)**	0.60 (0.44-0.83)**
2009-2010	1.01 (0.95-1.07)	0.63 (0.47-0.86)**	0.62 (0.46-0.83)**

^a^ Successfully traced vs not traced, ^b^ Patients who accessed alternative ARV sources vs. those who did not, ^c^ Patients with uninterrupted therapy vs. those who discontinued treatment. **^¥^**RR= Relative risk; *p < 0.05, **p < 0.01, ***p < 0.001

### Tracing outcomes

**Figure 1:**
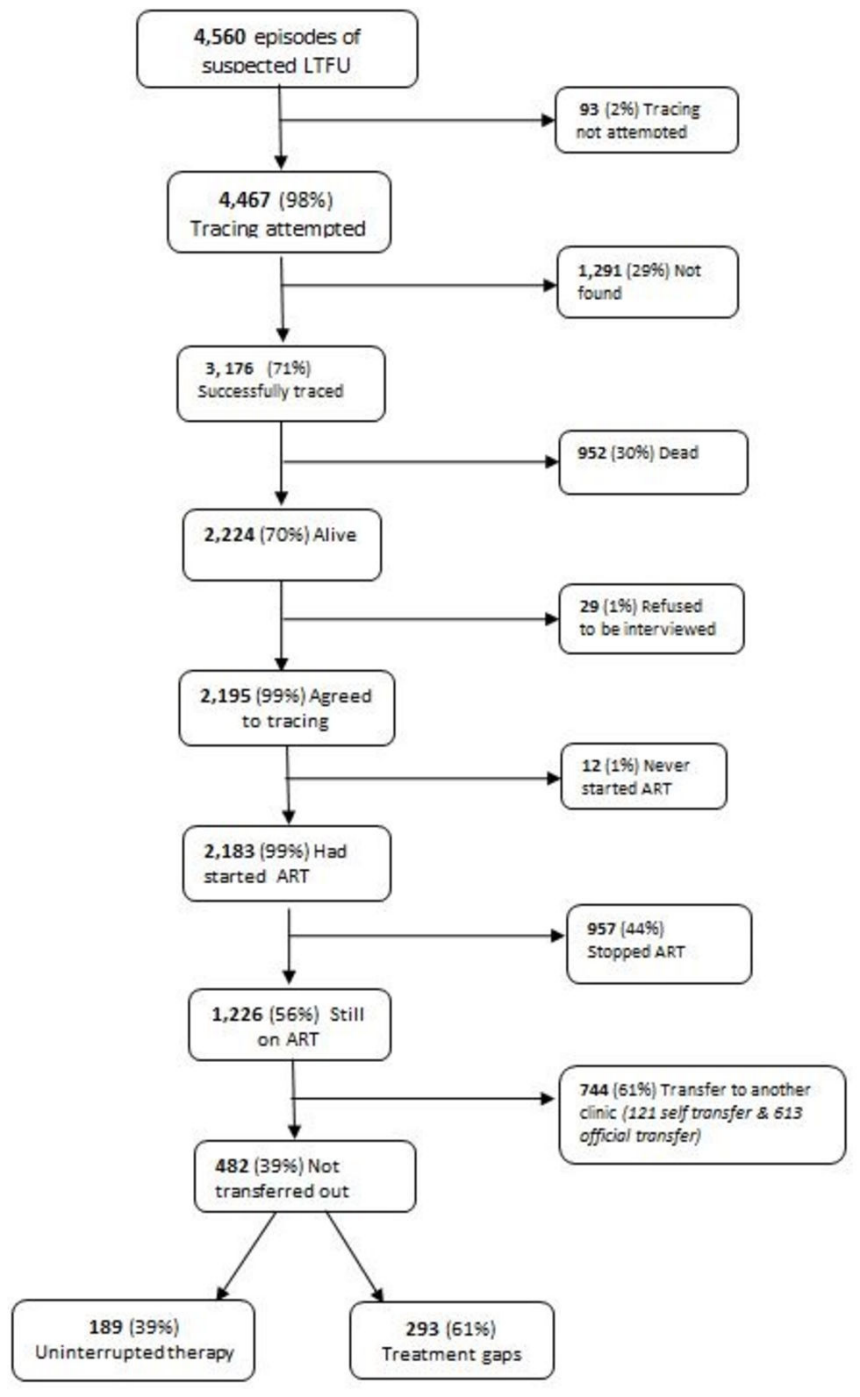
Flowchart of tracing outcomes for ART patients who run out of ARVs for at least 21 days between January 2006 and December 2010 at Martin Preuss Centre and Lighthouse clinics in Lilongwe, Malawi. LTFU = Lost to follow-up; Official transfer’ if transfer-out notes were available in the patient’s health book but not at the ART facility; or ‘self-transfer’ if the patient arranged the transfer independently; a patient could appear in more than one category during the study period. ‘Treatment gaps’ if a patient took none or fewer than the prescribed drugs before the interview date.

Figure **1** shows tracing outcomes for ART patients who ran out of ARVs for at least 21 days between January 2006 and December 2010 at Martin Preuss Centre and Lighthouse clinics. Of the 4,560 suspected LTFU cases, 1,384 (30%) could not be traced due to: incorrect/incomplete addresses or change of residence (1,291 (93%)); residence outside the clinics’ catchment areas (46 (3%)); or human resource shortages at either clinic (47 (4%)). Of the 3,176 successfully traced patients, 952 (30%) were dead and 2,224 (70%) were alive. Only 29 (1%) of the patients found alive refused to be interviewed. Of the 2,195 cases interviewed 2,183 (99%) had started ART according to phone-based self-reports or physical verification of health book or remaining pills during in-person interviews.

Of the 2,183 who had started ART, 1,226 (56%) reported still taking ARVs at the time of interview. Of those still on treatment, 744 (61%) reported receiving ART at another clinic: 121 had self-transferred and 623 had transferred officially but their clinic records were inaccurate. The remaining 482 (39%) were still in care at either Lighthouse or MPC clinics: 189 reported uninterrupted therapy despite missing an appointment and 293 reported treatment gaps.

### Access to and explanations for alternative ARV sources

**Figure 2:**
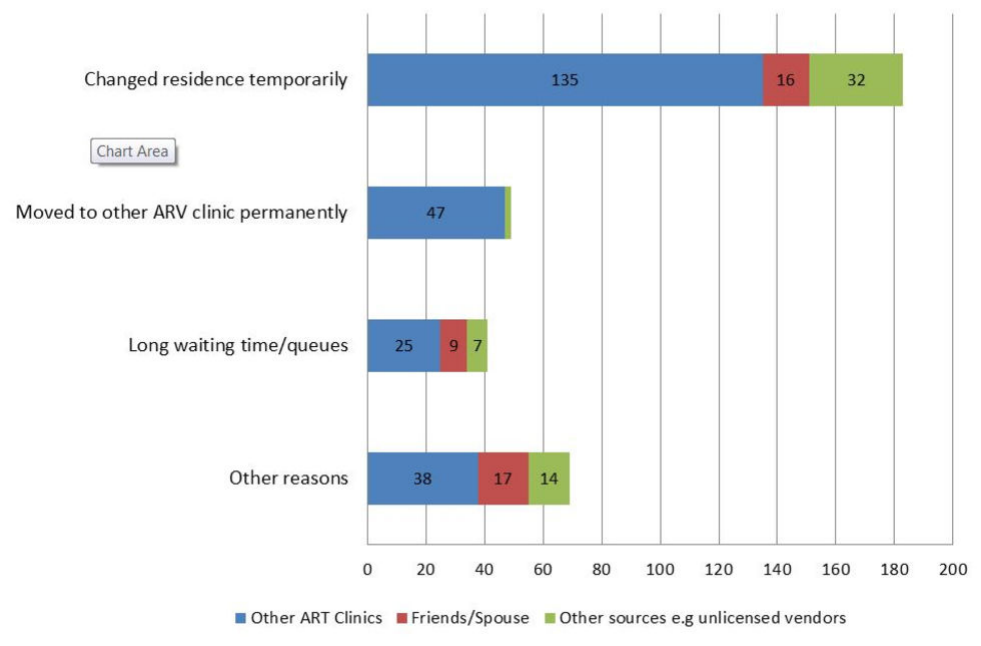
Reasons for collecting ARVs from other ART providers.

Among the 1,429 LTFU cases who reported self-transfer to another ART clinic, self-stopped ARVs, uninterrupted therapy or treatment gaps, we explored access to alternative sources of ART during the period they were assumed to be LTFU. No additional information was provided by 140 (10%) of patients. Of the remaining 1,289 patients, 311 (24%) collected ARVs from alternative sources; 204 (65%) received ARVs from another ARV clinic; 61 (20%) collected from drugs from other sources such as unlicensed vendors and 46 (15%) received ARVs from friends. Only 22 patients (7%) who collected ARVs from other sources had completely stopped ART before the time of interview. The most common reasons for collecting ARVs from other sources were temporary change of residence (65%), long waiting time/queues at the clinic (15%), moved to other ARV clinic permanently (18%), and other reasons (25%) (Figure **2**). LTFU patients who started ART in 2007 and after were less likely to access ART from alternative ART sources (2007: RR=0.65 (95% CI 0.49-0.87); 2008: RR =0.65 (95% 0.48-0.90); 2009-2010: RR= 0.63 (95% CI 0.47-0.86)) (Table **2**)

### Reasons for ART discontinuation

Of those who started ART and were found alive, 957 (44%) had stopped ART: 826 had stopped taking ART by themselves and 131 were stopped by a clinician as documented in patient’s health passport but not reflected in the electronic records. The median interval between last clinic visit and stopping ART was 30 days (IQR 0-38). LTFU patients who started ART in 2007 and after were less likely to have uninterrupted therapy than those starting before 2007 (2007: RR= 0.61 (95% CI 0.46-0.81); 2008: RR =0.60 (95% CI 0.44-0.83); 2009-2010: RR= 0.62 (0.46-0.83)).

All patients who were asked about alternative sources of ARVs were also asked if they stopped treatment during the considered LTFU period for any length of time. Of the 1,119 cases with treatment gaps and stopped ARVs, 179 (16%) cases did not provide a reason for ART discontinuation. Among the 940 cases with reasons, common explanations for discontinuation of ARVs were failure to remember taking ARVs [159 (17%)], feeling too weak/sick to go and collect ARVs [110 (12%)], travelling when ARVs ran out [431 (46%)] and lack of money for transport to go to the clinic [152 (16%)] (Table **3**). Men were more likely than women to discontinue ART due to travel (54% v 41%, *p*=0.001) while women were more likely to report lack of money for transport compared to men (22% v 14%, *p*=0.013).

**Table 3:** Reasons for discontinuing ART among patients stopped ART or had treatment gaps at Lighthouse and Martin Preuss Centre clinics^*^.

**Reasons for ART discontinuation**	**Total (1,119)**		**Stopped ART (n=826)**		**Treatment gaps** (**n=293**)	
Non- respondents	179		70		109	
Respondents	940		856		184	
Forgotten to take ARVs	159	17%	93	11%	66	36%
Suspected side effects of ARVs	43	5%	38	4%	5	3%
Very weak/sick	110	12%	91	11%	19	10%
Religious belief	65	7%	63	7%	2	1%
Travelled away	431	46%	363	42%	67	36%
Spouse did not approve taking ARVs	5	1%	5	1%	0	0%
Transport costs	152	16%	135	16%	17	9%
The clinic was not helping	42	4%	41	5%	1	1%
Feeling healthy	10	1%	9	1%	1	1%
Not ready to take ARVs for life	13	1%	13	2%	0	0%
Lack information about ARVs	10	1%	6	1%	4	2%
Work responsibilities	27	3%	26	3%	1	1%
Other reasons	225	24%	199	23%	26	14%

* Percentages are out of those who responded to each question; treatment gaps if a patient took none or fewer than the prescribed drugs before the interview date.

## Discussion

This large study from two high-volume public urban ART clinics found that 25% of patients suspected to be LTFU were actually found alive and on ART when traced. Of those, 61% had transferred to another ART clinic, 15% reported uninterrupted therapy using ARVs from other sources and 24% reported treatment gaps. About one fifth of traced patients reported stopping ART. Although most patients had stopped by themselves, some patients (3%) were stopped by a clinician and had erroneous patient records. Among the 18% of traced patients who stopped ART at some time, travel and financial constraints were the primary causes of discontinuation. These findings suggest both significant patient misclassification error and an underestimation of overall ART program retention. Due to the size of our patient population and the many years of operation, our results likely reflect similar patient misclassifications and ART program outcomes at the national level. Using these results, we provide insight into interventions for improving retention in ART clinics and suggest improvements to estimating retention.

First, our findings suggest that statistical methods to account for mortality, treatment gaps and uninterrupted therapy among patients classified as LTFU are needed. Approaches to correct mortality [6] [4] and patients who self-transfer to another clinic [15] for LTFU estimates exist; however, there are no methods that approximate uninterrupted therapy and treatment gaps among patients considered LTFU. As uninterrupted therapy and treatment gaps comprise a sizeable proportion of those identified as LTFU in our study, incorrectly accounting for these outcomes may lead to underestimation of retention. For example, out of a total of 391,300 patients alive on ART by 30 September 2012 in the Malawi National ART cohort, approximately 90,000 patients were reported to be LTFU [16]. Applying the proportion of presumed LTFU found at Lighthouse and MPC clinics to the national cohort data, almost 22,000 patients would be incorrectly categorized as disengagement of care. Even if the proportion of patients who are true LTFU at the Lighthouse and MPC clinics are slightly lower than the national average, the incorrect classification of LTFU patients as being completely off treatment may lead to inaccurate drug forecasting and underestimation of drugs and commodities at the national level. Considering the significant resource constraints of most ART clinics in Malawi and in the region, similar tracing programs of LTFU patients as part of routine monitoring and evaluation using the B2C model are impractical. However, using our findings to revise LTFU figures to reflect numbers of patients with uninterrupted therapy or short-term treatment interruptions may prove advantageous for program evaluation and forecasting. This recalculation becomes more critical as increasing ART cohorts in Malawi and other high burden countries will amplify this misclassification error and result in program reporting bias.

Second, our study has implications for improvements at the clinic level. We found that a substantial proportion of patients initially considered LTFU were actually alive and collecting ART from friends or other sources outside ART clinics, suggesting the need for both clinic-based retention interventions and improvements in data quality. One frequently cited reason for collecting ART from friends or other sources was travel; this reason was more frequently cited by men who often migrate for work. Although patient travel is hard to predict, for patients who inform clinic staff, increasing the number of pills prescribed per visit to meet patient needs is advisable. In Malawi, national policy notes that all documented ART patients may collect a one-month, emergency supply of ARVs from any other ART clinic – a policy specifically designed to help address patient travel needs. However, clinicians and patients may not know about this possibility, dramatically reducing the effectiveness of this proactive policy. Educational efforts aimed at reminding clinic staff and ART patients about this policy, recognition of seasonal harvest periods which rely heavily on male labor, and better documentation of policy application in patient health passports are clearly needed to meet patient needs and maintain accurate records. A greater challenge may be presented by patients who collect drugs from friends, family or other sources when they travel: their alternative-source ARVs might be different from those prescribed at the ART clinic, potentially reducing drug effectiveness, aiding the development of side effects, or increasing potential for drug resistance. Furthermore, waiting times and long queues were also noted as a reason for missing appointments. Lighthouse and MPC are the largest ARV providers in the central region of Malawi. Almost 17% of ART patients in Lilongwe district access ART at these two clinics, mainly because Lighthouse is a referral ART clinic and MPC is located close to a transport hub easing travel to clinic. In an internal study on clinic waiting times (unpublished data), it was found that both clinics review around 600 patients a day and have patient waiting times that range from 78 to 87 minutes per patient. Although this waiting time is lower than the average waiting times reported by other similar clinics [17] [18], Lighthouse and MPC are considering options to further reduce waiting times by dispensing 2 to 3 months drug refill periods for stable patients in order to minimise clinic visits, scheduling visits over weekends, making time-specific appointments for each patient in a day and providing ART in the community through community based organisations. Lastly, misclassifications also indicate issues in the data quality at Lighthouse and MPC as well as highlight the need for ART clinics to improve communication and transfers between sites. Almost 23% of those successfully traced were accessing ART from other clinics while being misclassified as LFTU at their previous ART clinic. ART clinics need to establish data linkages through which information of patients that transfer can be shared.

Third, although significant proportions of patients considered to be LTFU are dead or transferred to other clinics or are still on ART, individual-level interventions are necessary to reduce ART discontinuations as any treatment interruption could increase the development of drug resistance, with both individual- and population-level repercussions [19]. Decreasing treatment interruptions requires specific, targeted interventions that combine increased prevention of LTFU by providers with improved responses to patient challenges. The most commonly cited reasons for ART discontinuation are financial constraints and travel, similar to findings from previous studies [20] [21]. These findings differ by gender; more men reporting travel issues while more women reported financial constraints. Patients who started ART in 2007 and after were less likely to have uninterrupted therapy possibly because, by this time, there were a number of ART clinics in the district. The findings suggest that strategies for improving ART retention must take into account the dual responsibilities of both providers and patients and that interventions may need to differ by gender. On the provider side, ART programs should further decentralize ART clinics to reduce patient transport costs. Although these efforts could benefit all patients, bringing ARVs into the community may provide additional benefits for female patients who may not control household finance and, therefore, may encounter more problems securing money for travel costs [22]. Additionally, as mentioned above, ART programs should consider providing larger quantities of ARVs per visit for stable patients to reduce the number of clinics visits, reducing transport costs, decrease waiting times and better supply those with unexpected travel. Lastly, a previous study noted patient fear or concerns about provider reactions to their return to care as reason for ART discontinuation [23]. Although not specifically addressed in our study, additional efforts to sensitize healthcare staff to be more welcoming when patients return after missing or delaying appointments is critical to the success of any intervention.

Our study should be considered with the following limitations. First, we used routine program data collected from our patient tracing program: some information was missing. Additional reasons for patient discontinuation or treatment interruption were not available, limiting the scope of the study. Moreover, there may be differences in proportion of LTFU with uninterrupted therapy and treatment gaps between patients who are LTFU in urban ART clinics compared with those from rural or private settings. Patients in rural ART clinics may have lower mobility and fewer alternative sources of ART than their urban peers. Moreover, self-reported data may reflect social desirability bias so the true number of patients off treatment may be higher. Despite these limitations, we believe that our large sample size enables application of our findings to other large urban clinics and makes our recommendations useful to inform policy and the national ART program in Malawi and other comparable settings.

Overall, although patients that are considered to be LTFU comprise a sizeable proportion of true ART patient outcomes, the category of LTFU includes a large proportion of patients with uninterrupted therapy and treatment gaps that may bias ART retention estimates at both the clinic and national levels. While simultaneously making statistical adjustments to LTFU estimates to account for actual treatment gaps and non-interrupted therapy, clinics and providers could potentially expand clinic hours, increase ARV allocations for stable patients, or work to further decentralize ARV distribution locations to prevent LTFU. In combination, these policy and program efforts could increase the accuracy of ART program reporting and improve patient retention in care, making significant contributions to improved management of ART programs at the clinic and national levels.
